# Population Diversity of *Campylobacter jejuni* in Poultry and Its Dynamic of Contamination in Chicken Meat

**DOI:** 10.1155/2015/859845

**Published:** 2015-10-12

**Authors:** Francesca Marotta, Giuliano Garofolo, Guido Di Donato, Giuseppe Aprea, Ilenia Platone, Silvia Cianciavicchia, Alessandra Alessiani, Elisabetta Di Giannatale

**Affiliations:** Istituto Zooprofilattico Sperimentale dell'Abruzzo e del Molise “G.Caporale”, National Reference Laboratory for Campylobacter, 64100 Teramo, Italy

## Abstract

This study aimed to analyse the diversity of the *Campylobacter jejuni* population in broilers and to evaluate the major source of contamination in poultry meat. Eight rearing cycles over one year provided samples from three different broiler farms processed at the same slaughterhouse. A total of 707  *C. jejuni* were isolated from cloacal swabs before slaughter and from the breast skin of carcasses after slaughter and after chilling. All suspected *Campylobacter* colonies were identified with PCR assays and *C. jejuni* was genotyped by sequence analysis of the *flaA* short variable region (SVR) and by pulsed-field gel electrophoresis (PFGE) using *SmaI* enzyme. Phenotypic antibiotic resistance profiles were also assayed using minimal inhibitory concentration (MIC). The flocks carried many major *C. jejuni* clones possibly carrying over the rearing cycles, but cross contamination between farms may happen. Many isolates were resistant to fluoroquinolones, raising an issue of high public concern. Specific *Campylobacter* populations could be harboured within each poultry farm, with the ability to contaminate chickens during each new cycle. Thus, although biosecurity measures are applied, with a persistent source of contamination, they cannot be efficient. The role of the environment needs further investigation to better address strategies to control *Campylobacter*.

## 1. Introduction

*Campylobacter* is the most common cause of bacterial gastroenteritis in Europe. The incidence of human campylobacteriosis is increasing worldwide, as well as the number of isolates resistant to fluoroquinolones which are one of the primary classes of antimicrobials used to treat* Campylobacter* infection in human therapy and thus considered of high public concern [1]. In the European Union,* Campylobacter* is still the most commonly reported cause of bacterial foodborne illness with a notification rate of 55.49 cases per 100,000 of population in 2012 [2]. Poultry is a natural reservoir of* Campylobacter* species, constituting the most important source of human infection. The consumption of undercooked poultry meat or the mishandling of raw poultry products is considered to be the main risk factors associated with human campylobacteriosis [3–5].

The prevalence of* Campylobacter* in broiler chicken flocks ranges from 3 to 90% depending on their location [6, 7] and the isolation rates within positive flocks at slaughter are high (around 80%) [8–10]. Recent studies have reported that the prevalence of* Campylobacter* in retail chicken products ranges from 90 to 100% across several countries [11, 12].* Campylobacter* colonization in chickens takes place at poultry farms, approximately 7 days after hatching [13], while widespread carcass contamination occurs at the slaughterhouse, especially from cross contamination by intestinal contents after the evisceration phase or from dirty surfaces [14]. Nevertheless, there have been few studies on the contamination of poultry carcasses from the farm through the entire production chain up to the retailer [15, 16] so the contamination routes in broiler flocks are still unknown.

The objective of the present study was to perform a comprehensive molecular characterization of* C. jejuni* isolated from poultry on the farm and during the slaughter process. Different typing methods, such as PFGE and* flaA*-SVR sequencing, will be used to trace the contamination of chicken products and to investigate the potential of specific isolates to persist or be predominant in the poultry production. PFGE has been successfully applied to track* Campylobacter* during poultry production [16–18] and, together with* flaA*-SVR sequencing, it represents a highly discriminatory method for a better understanding of* Campylobacter* population structures. In addition, antibiotic susceptibility will also be investigated to determine the resistance pattern of* Campylobacter* that spread from chickens to humans along the poultry food chain, although the correlation between resistant bacteria in people and the use of antibiotics in feed is still a matter of debate [19].

## 2. Materials and Methods

### 2.1. Broiler Farms

Three different broiler farms (A, B, and C), randomly selected in the Abruzzo region of central Italy and spaced about 40 kilometres apart in a narrow zone, were enrolled in the study. The farms were managed similarly as part of the same integrated broiler company under good hygiene practices, rearing flocks of 40,000–60,000 birds with an average age at slaughter of 38–42 days.

### 2.2. Experimental Set-Up

Four different flocks were monitored on farm A, and two flocks each on farms B and C, amounting to a total of eight different rearing cycles under study between July 2011 and July 2012 with detailed dates shown in Table 1. For each flock, one day before slaughter, 50 different chickens, individually identified by leg rings, were randomly chosen and cloacal swabs taken (F), which were transported immediately to the laboratory using Ames transportation medium. The following day, the birds were transported 50 kilometres to the company abattoir, where samples were taken after slaughter (S) and after the chilling process (C). Samples S and C consisted of breast skin sampled under aseptic conditions, which were transported to the laboratory in a portable cooler at 2–4°C for immediate processing. The flocks tested were the first to be slaughtered on these days, using a slaughter line disinfected after the last batch processed on the previous day.

### 2.3. Culture Conditions and PCR Assays

A total of 1,720 samples were processed during the whole project and* Campylobacter* was recovered from carcass samples after the enrichment and the enumeration phases, according to parts 1 and 2, respectively, of the NF EN ISO 10272 standard procedure [20, 21]. The isolates were cultured on Columbia blood agar and incubated at 42°C for 48 h in a microaerophilic atmosphere. After a preliminary phenotypic characterization, suspected colonies were confirmed as thermotolerant* Campylobacter* and identified to species level using a multiplex PCR, as described previously by Di Giannatale et al. [22]. Genomic DNA was extracted using a Wizard genomic DNA purification kit (Promega, Madison, WI, USA). Isolates were stored in a Microbank (Pro-Lab Diagnostics Canada, Richmond Hill, ON, Canada) at 80°C until further analysis.

### 2.4. PFGE

Pulsed-field gel electrophoresiswas performed according to the 2009 U.S. PulseNet protocol for* Campylobacter* [23]. Briefly, bacteria previously identified as* Campylobacter *by PCR were subcultured on Columbia blood agar at 42°C for 2 days in a microaerophilic atmosphere and embedded in agarose blocks (Seakem Gold Agarose, Lonza, Rockland, ME, USA). The blocks were then lysed, washed, digested with 25 U of SmaI restriction endonuclease (Promega, Milan, Italy) at 25°C overnight, and subjected to pulsed-field electrophoresis in 1% agarose gel (Seakem Gold Agarose). PFGE was performed using a Chef Mapper XA (Bio-rad Laboratories, Hercules, CA, USA) and* Salmonella* serovar* Branderup* H9812 was used as standard molecular weight size. After electrophoresis, the gel was stained with Sybr Safe DNA gel stain (Invitrogen, Waltham, MA, USA) and photographed with a transilluminator (Alpha Innotech, San Leandro, CA, USA). For image analysis, Bionumerics v. 6.6 software (Applied Maths, Sint Martens Latem, Belgium) was used to identify the clusters of closely related or identical patterns. Pair comparisons and cluster analyses were carried out using the Dice correlation coefficient (position tolerance, 1.0%) and the unweighted pair group mathematical average (UPGMA) clustering algorithm. PFGE clusters were arbitrarily defined at a similarity level of 60% [24]. Untypeable isolates were not included in the analysis.

### 2.5. *flaA* SVR Sequencing

Typing was performed by amplifying the* flaA*-SVR using primers as described by Nachamkin et al. [25], followed by sequencing of the PCR product. Amplification products were verified by gel electrophoresis. PCR products were purified by using ExoSAP-IT reagent (GE Healthcare, Santa Clara, CA, USA) and sequenced using the BigDye Terminator v.3.1 Cycle sequencing kit (Applied Biosystems, Darmstadt, Germany) according to the manufacturer's instructions. After sequencing, DNA was purified with ethanol precipitation using the Agencourt CleanSEQ kit (Beckman Coulter, Brea, CA, USA). Sequencing products were analysed with a Genetic Analyzer 3500 (Life Technologies, Paisley, UK). The nucleotide sequences were compared with the* C. jejuni flaA *database (http://pubmlst.org/campylobacter/) and allele numbers were assigned accordingly. Confirmed sequences were aligned using MEGA 4 software [26]. For new* flaA*-SVR alleles, DNA trace files were submitted to the database administrator for confirmation. The peptide sequences were translated from the DNA sequences and named according to the Oxford database available at http://pubmlst.org/campylobacter/ The genetic diversity and the comparison between the molecular methods were determined using the Simpson's diversity index (SDI) and the adjusted Rand index (aRI) via the online tool available at the Comparing Partitions website (http://darwin.phyloviz.net/ComparingPartitions/index.php?link=Home).

### 2.6. Antimicrobial Susceptibility

*Campylobacter* susceptibility to antibiotics was evaluated using the microdilution method by the “Sensititre” automated system (TREK Diagnostic Systems/Biomedical Service, Venice, Italy). Colonies were harvested in Columbia agar for 24 hours, inoculated in Mueller Hinton Broth supplemented with blood, and dispensed into Eucamp microtiter plates (TREK Diagnostic Systems/Biomedical Service), containing known scalar concentrations of the following antibiotics: gentamicin (0.12–16 *μ*g/mL), streptomycin (1–16 *μ*g/mL), ciprofloxacin (0.06–4 *μ*g/mL), tetracycline (0.25–16 *μ*g/mL), erythromycin (0.5–32 *μ*g/mL), nalidixic acid (2–64 *μ*g/mL), and chloramphenicol (2–32 *μ*g/mL). The plates were then incubated at 42°C in a microaerophilic atmosphere for 24 hours.* C. jejuni* NCTC 11351 was included for the quality control in the MIC test.

## 3. Results

### 3.1. *Campylobacter* Prevalence

*Campylobacter *spp. was isolated in 1,081 of the samples. Further differentiation within the* Campylobacter* genus was obtained by PCR, resulting in 374* C. coli* and 707* C. jejuni*. The isolates were recovered from the different sources as follows: 281* C. jejuni* and 56* C. coli* from broiler flocks from the three farms, 366* C. jejuni* and 248* C. coli* from carcasses processed in the slaughterhouse, and 60* C. jejuni* and 70* C. coli* after chilling. At farm level, the prevalence of* C. jejuni* (65.77%) was significantly higher (*P* < 0.05, *χ* ^2^ test) than* C. coli* isolates (12.62%). All the flocks investigated from the different farms were positive for* Campylobacter* with high rates of prevalence, ranging from 58 to 90% of positive chicken (data not shown). In contrast, after chilling, the prevalence of* C. coli* (39.10%) was significantly higher (*P* < 0.05, *χ* ^2^ test) than* C. coli* groups at farm level (12.62%).

### 3.2. Typing

**Figure 1:**
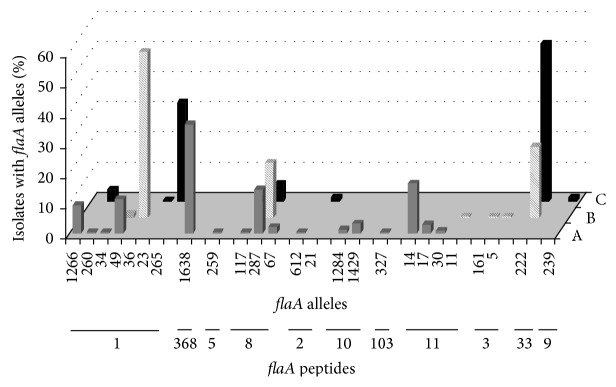
Distribution of* flaA* alleles and peptides (shown on the *x*-axis) from farms A, B, and C.

From farm A, 249 samples of* C. jejuni* were isolated during all four sampling periods (Table 1). Molecular investigation of the short variable region of the flagella revealed 16 different nucleotide types that corresponded to eight different peptide types (Table 1). Each flock was characterized by 5 to 9 different* flaA* types with one type predominant. In 6 instances, the same* flaA* type was recovered from different samples. The* flaA* type 1638 was isolated from all four rearing cycles, while* flaA* types 14 and 17 and* flaA* types 30, 49, 67, and 287 were isolated from 3 and 2 cycles, respectively (Table 1). From the analysis of isolates from the single flock of farm A, only 6 (4.38%) out of 137* C. jejuni* isolated in the slaughterhouse did not belong to* flaA* types recovered from the farm. These remaining isolates showed the same fla type as those from the farm (Table 2). At a 60% similarity level, the PFGE clustering analysis revealed a high diversity within the isolates, grouping most of the isolates in three major clusters. The first cluster included 33* C. jejuni *isolated from three rearing cycles (12.12.2011–10.05.2012–17.07.2012); the second cluster contained 130* C. jejuni* isolated from all cycles analysed (12.12.2011–12.02.2012–10.05.2012–17.07.2012); the last cluster included 21 isolates from two cycles (12.12.2011–17.07.2012). All* C. jejuni* isolates in the three PFGE clusters were detected at farm, slaughter, and postchilling level. At a 100% similarity level, a dendrogram combining the data from farm A resulted in 56 different PFGE pulsotypes. Four pulsotypes comprised 49.57% (117/236) of the* C. jejuni* isolates from farm A, while 35 of the 56 pulsotypes included only a single* C. jejuni* isolate (Table 3). The polymorphisms resulting from the PFGE were higher than* flaA* typing with an SDI of 0.84 against 0.79; nevertheless the agreement between the methods resulted in an aRI of 0.44. A total of 225 strains of* C. jejuni* were isolated from farm B, 115 in the summer (19.09.2011) and 110 in the autumn (17.11.2011). Molecular investigation of the flagella determined seven different nucleotide types corresponding to five different peptide types (Table 1). Only* fla* type 36 was recovered in both rearing cycles analysed. All the isolates collected after chilling showed* fla* types previously detected in the live chicken. Six isolates out of 123 collected from the slaughterhouse featured four* fla* types (49, 11, 161, and 5) that were different from those collected on the farm (Table 2). At a 60% similarity level, the PFGE clustering showed a high variability with four major clusters. The first cluster included 80* C. jejuni *isolated from two rearing cycles (19.09.2011–17.11.2011) obtained at farm, slaughter, and postchilling phases; the second cluster contained 96* C. jejuni* from one flock (19.09.2011) but they were present in all the phases analysed; the third cluster included 26 isolates from one flock (17.11.2011) at farm and slaughter level; the last cluster included 16 isolates from two rearing cycles (19.09.2011–17.11.2011) obtained at the farm and slaughter phases. At a 100% similarity level, all the* C. jejuni* from farm B were clustered in 39 different pulsotypes with four that comprised 53.73% of the isolates and 22 pulsotypes represented by a single isolate (Table 3). The polymorphisms of PFGE showed an SDI of 0.927, higher than the* fla* typing value of 0.591, but agreed well with an aRI of 0.819. A total of 219* C. jejuni* were recovered from farm C, 126 in the winter (31.03.2012) and 93 in the spring (12.06.2012). The* fla *SVR sequencing identified seven* fla *SVR sequences, corresponding to five peptide alleles (Table 1). Both samplings from this farm revealed the* fla* type 287 (Table 1). From the slaughterhouse, 4 out of 219 isolates provided two* fla* types not present in those* C. jejuni* from the cloacal swabs (Table 2). At a 60% similarity level, the PFGE clustering showed a high variability with three major clusters. The first cluster included 49* C. jejuni *isolated from one rearing cycle (12.06.2012) collected during the farm, slaughter, and postchilling phases; the second cluster contained 11* C. jejuni* isolated in two rearing cycles (31.03.2012–12.06.2012) during the farm and slaughter phases; the third cluster included 134* C. jejuni* isolated in two rearing cycles (31.03.2012–12.06.2012) during the farm, slaughter, and postchilling phases. At a 100% similarity level, the samples from farm C were divided into 60 different pulsotypes, with three of them comprising 30.85% (58/188) of the isolates and 43 pulsotypes represented by a single isolate (Table 3). For PFGE, the SDI for this cycle was 0.944 while that for the* fla* type was 0.627 and the aRI displayed a fairly high value of 0.864. The distribution of* flaA* alleles and peptides isolates in the three farms A, B, and C is summarized in Figure 1. Sixty-nine isolates (9.76%) were untypeable with PFGE, appearing to be a case of DNA smearing rather than restriction.

### 3.3. Antimicrobial Susceptibility Tests

MIC and antimicrobial resistance of all* Campylobacter* isolates tested in this study are presented in Table 4. The MIC test revealed that 90% of the isolates were resistant to quinolones (NAL and CIP), but 98% were susceptible to chloramphenicol and streptomycin and 99% susceptible to gentamicin. Notably, 64% of the* Campylobacter* showed resistance to tetracycline, 18% showed resistance to erythromycin, and a few isolates were resistant to other antimicrobials such as chloramphenicol (1.2%), streptomycin (1%), and gentamicin (0.3%). Furthermore, resistance to erythromycin and tetracycline antimicrobials was significantly more frequent in* C. coli* compared with* C. jejuni *(*P* < 0.05, *χ* ^2^ test), whereas no differences were observed for the remaining antibiotics.

## 4. Discussion

**Figure 2:**
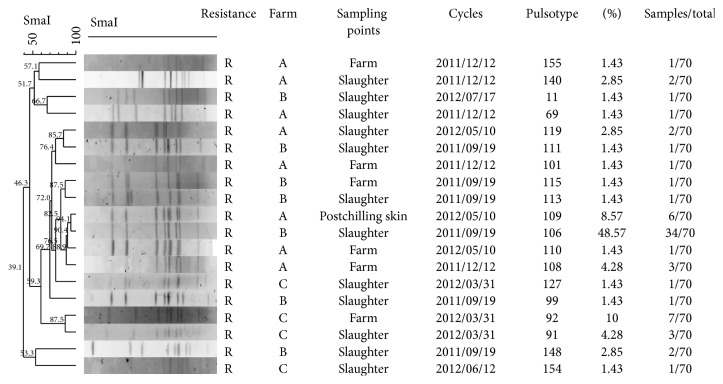
Dendrogram of* C. jejuni* SmaI PFGE patterns isolated in the three farms characterized by* flaA* allele 287 and antimicrobial resistance to fluoroquinolones.

Over the last five years, campylobacteriosis has become more prevalent in Europe.* Campylobacter* is found mostly in chicken meat with poultry and poultry farms playing a key role in the epidemiology of human infection [27, 28]. In Italy, a European survey showed a prevalence of* Campylobacter-*colonized broiler batches of 63.3% [9]. Similar prevalence levels in Italy (60%) have recently been obtained by other studies [29, 30]. The present study aimed to analyse the diversity of the* C. jejuni* population in poultry and to monitor the contamination process throughout the farm, slaughter, and postchilling phases. The results have shown a very diverse* C. jejuni* population, even though only three broiler farms from a narrow area were evaluated. A total of 25* flaA*-SVR types and 11* flaA *peptides were identified among the numerous isolates that were analysed, demonstrating the presence of a heterogeneous population. This is also supported by previous studies where isolates from different continents were assessed by* flaA* SVR typing revealing a similar degree of diversity [31, 32]. Interestingly, we found a high individual prevalence of* Campylobacter, *in common with other studies [10]. Tracing back the* Campylobacter* for each flock showed that the major source of chicken meat contamination remains the flock itself. In the present study, only 10% of the isolates from the abattoir were distinguishable from the live chicken isolates, showing that there were few cases of contamination during slaughtering. Frequently, the most common* fla* types in live chickens were also the most common genotypes in the processed carcasses and this confirms results reported in previous studies [6, 10, 33, 34]. In a context where all flocks are contaminated, it seems that the slaughterhouse does not play an important role in carcass contamination. However, the situation completely changes when* Campylobacter*-free flocks meet contaminated flocks at the abattoir. It is therefore sound practice for contaminated poultry flocks to be slaughtered at the end of the working day to contain the cross contamination among the flocks. So diagnostic systems must be able to detect* Campylobacter* and distinguish uncontaminated from contaminated flocks. The potential of* Campylobacter* to carry over to succeeding rearing cycles would indirectly suggest its ability to survive within the broiler farm. A comparison of isolates from different samplings for farm A showed that seven* fla* alleles (83.94%) recurred over a period of almost 8 months. The overlaps of* fla* genotypes were minor for farms B and C, probably because of the short length of monitoring undertaken, although communities were also demonstrated by the carryover of alleles 36 and 287. To strengthen these findings, we also found that PFGE clustering at 60% of similarity grouped isolates from different rearing cycles. These isolates fell into the same PFGE cluster and featured the same* fla* allele suspected to be stable over time thus indicating that some isolates were successful in the broilers. The* fla* SVR analysis also showed that 38.57% of the isolates shared the same* fla* alleles among the three farms (Figure 1), although a limited correlation between the farms could be argued.* fla* allele 287, in particular, revealed a PFGE clustering, supporting the hypothesis that all the isolates were strongly related, independently of the farms (Figure 2). This could be explained by cross contamination, probably caused by objects that might transport* Campylobacter *within the broiler houses most likely during the thinning process. In the present study, the farms were managed as part of a vertically integrated supply chain. Generally, feed mills, breeding farms, hatcheries, and slaughterhouses are owned by the same company and it is probable that the same catching crew could cross contaminate the farms by using unclean crates. Monitoring these practices very thoroughly is required to better address these types of problem. Antibiotic resistance has been a long-standing problem in the field of human and veterinary medicine [31, 35–40] generally related to the indiscriminate use of antibiotics in prophylaxis and therapy or as a growth promoter [39]. Comparative studies of isolates from different geographical areas show a steady and alarming increase in resistance, even to the next generation molecules [31, 32, 37, 40–42]. Particularly worrying is the increase in the frequency of resistance against fluoroquinolones, particularly ciprofloxacin [10], which was confirmed in our study (90%). Moreover our results on resistance against nalidixic acid (90%) and tetracycline (64%) agreed with those in the EFSA Report [42] and other studies [31, 43], confirming this increasing trend. The susceptibility against chloramphenicol (1.2%), streptomycin (1%), and gentamicin (0.3%) could be probably attributable to the lack of extensive use of these drugs in Italy.

## 5. Conclusions

This study has revealed the usefulness of molecular methods for tracing* Campylobacter* contamination in the poultry supply chain. These data have provided more information on the presence of* Campylobacter* clones that have adapted well to poultry and can survive on the farms. The question arises whether* Campylobacter* has an ecological niche that permits its survival. Several hypotheses have been debated but no data are available to evaluate water supplies and vectors such as flying insects or rodents as potential risk factors involved in the mechanism of contamination [6, 44]. Our results showed a highly diverse* C. jejuni* population in poultry, suggesting that its introduction or reintroduction on the farm may originate from different sources. Since the main source of poultry meat contamination was confirmed to be the flock, it is reasonable to suggest that* Campylobacter*-free meat could be achieved by reducing its prevalence at farm level.

## Figures and Tables

**Table 1:** Distribution of *flaA* and peptide types according to rearing cycle from farms A, B, and C.

	Peptide type	*flaA type *	12/12/2011	12/02/2012	10/05/2012	17/07/2012	Proportion	Number of strains
Farm A	1	1266	—	23 (24.46%)	—	—	9.23%	23
		260	1 (1.38%)	—	—	—	0.4%	1
		34	1 (1.38%)	—	—	—	0.4%	1
		49	—	—	1 (2.94%)	27 (55.10%)	11.24%	28
	368	1638	1 (1.38%)	67 (71.27%)	3 (8.82%)	19 (38.77%)	36.14%	90
	5	259	—	—	—	1 (2.04%)	0.4%	1
	8	117	—	—	—	1 (2.04%)	0.4%	1
		287	19 (26.38%)	—	17 (50.00%)	—	14.45%	36
		67	—	1 (1.03%)	4 (11.76%)	—	2%	5
	2	612	1 (1.38%)	—	—	—	0.4%	1
	10	1284	—	—	3 (8.82%)	—	1.21%	3
		1429	8 (11.11%)	—	—	—	3.21%	8
	103	327	1 (1.38%)	—	—	—	0.4%	1
	11	14	37 (51.38%)	1 (1.03%)	3 (8.82%)	—	16.46%	41
		17	3 (4.16%)	2 (2.12%)	2 (5.82%)	—	2.81%	7
		30			1 (2.94%)	1 (2.04%)	0.8%	2
	Total		**72**	**94**	**34**	**49**		**249**

			19/09/2011	17/11/2011	—	**—**		

Farm B	1	36	18 (15.65%)	106 (96.36%)	—	—	55.11%	124
		49	—	3 (2.72%)	—	—	1.33%	3
	11	11	1 (0.86%)	—	—	—	0.44%	1
	3	161	1 (0.86%)	—	—	—	0.44%	1
		5	—	1 (0.90%)	—	—	0.44%	1
	33	222	53 (46.08%)	—	—	—	23.55%	53
	8	287	42 (36.52%)	—	—	—	18.22%	42
	Total		**115**	**110**	**—**	**—**		**225**

			31/03/2012	12/06/2012	**—**	—		

Farm C	1	260	—	9 (10%)	—	—	4.1%	9
		23	—	1 (1.11%)	—	—	0.5%	1
		265	—	72 (80%)	—	—	32.9%	72
	8	117	3 (2.32%)	—	—	—	1.4%	3
		287	11 (8.52%)	2 (2.22%)	—	—	5.9%	13
	2	21	—	3 (3.33%)	—	—	1.4%	3
	33	222	1158 (89.14%)	—	—	—	52.5%	115
	9	239	—	3 (3.33%)	—	—	1.4%	3
		Total	**129**	**90**	**—**	**—**		**219**

**(a):** 

	*flaA type *	12/12/2011			12/02/2012			10/05/2012			17/07/2012		
		F	S	C	F	S	C	F	S	C	F	S	C
Farm A	1266	—	—	—	19	4	—	—	—	—	—	—	—
	260	—	1	—	—	—	—	—	—	—	—	—	—
	34	—	1	—	—	—	—	—	—	—	—	—	—
	49	—	—	—	—	—	—	—	1	—	7	20	—
	1638	—	1	—	16	48	3	1	2	—	2	15	2
	259	—	—	—	—	—	—	—	—	—	—	1	—
	117	—	—	—	—	—	—	—	—	—	1	—	—
	287	9	10	—	—	—	—	5	11	1	—	—	—
	67	—	—	—	—	1	—	3	1	—	—	—	—
	612	—	1	—	—	—	—	—	—	—	—	—	—
	1284	—	—	—	—	—	—	2	1	—	—	—	—
	1429	2	6	—	—	—	—	—	—	—	—	—	—
	327	—	1	—	—	—	—	—	—	—	—	—	—
	14	15	22	—	—	1	—	1	2	—	—	—	—
	17	2	1	—	—	1	1	2	—	—	—	—	—
	30	—	—	—	—	—	—	—	—	1	—	1	—
	Total	**28**	**44**	**0**	**35**	**55**	**4**	**14**	**18**	**2**	**10**	**37**	**2**

**(b):** 

	*flaA type *	19/09/2011			17/11/2011		
		F	S	C	F	S	C
Farm B	36	3	13	2	45	49	12
	49	—	—	—	—	3	—
	11	—	1	—	—	—	—
	161	—	1	—	—	—	—
	5	—	—	—	—	1	—
	222	21	29	3	—	—	—
	287	6	26	10	—	—	—
	Total	**30**	**70**	**15**	**45**	**53**	**12**

**(c):** 

	*flaA type *	31/03/2012			12/06/2012		
		F	S	C	F	S	C
Farm C	260	—	—	—	3	6	—
	23	—	—	—	—	1	—
	265	—	—	—	34	38	—
	117	—	3	—	—	—	—
	287	5	5	1	1	1	—
	21	—	—	—	—		3
	222	36	60	19	—	—	—
	239	—	—	—	2	1	—
	Total	**41**	**68**	**20**	**40**	**47**	**3**

F = cloacal swabs; S = slaughterhouse line; C = postchilling phase.

**Table 3:** Distribution of PFGE pulsotypesat 100% similarity according to sampling point from farms A, B, and C.

	PFGE pulsotypes	Cycles	Major PFGE pulsotype	F (Number of isolates/total number of samples)	S (Number of isolates/total number of samples)	C (Number of isolates/total number of samples)
Farm A	56	12.12.2011	A	(10/236)	(9/236)	(7/236)
			D	(7/236)	(5/236)	(1/236)
		22.02.2012	B	(22/236)	(31/236)	(5/236)
		17.07.2012	B	(5/236)	(1/236)	(1/236)
			C	(8/236)	(5/236)	(0/236)

Farm B	39	19.09.2011	E	(16/214)	(18/214)	(2/214)
			F	(5/214)	(12/214)	(8/214)
		17.11.2011	G	(10/214)	(14/214)	(0/214)
			H	(17/214)	(8/214)	(5/214)

Farm C	60	31.03.2012	I	(0/188)	(2/188)	(0/188)
			L	(14/188)	(5/188)	(0/188)
			M	(0/188)	(5/188)	(12/188)
		12.06.2012	I	(1/188)	(9/188)	(0/188)

Sampling point: F = cloacal swabs; S = slaughterhouse line; C = postchilling phase.

**Table 4:** Antimicrobials, dilution ranges, and cut-off values used for MIC determination of *Campylobacter*.

Antimicrobials	MIC breakpoints (*µ*g/mL)		Distribution % of MIC (*µ*g/mL)											MIC_50_ (*µ*g/mL)	MIC_90_ (*µ*g/mL)	Number of resistant isolates (%)	Number of resistant *C. jejuni * (%)	Number of resistant *C. coli * (%)
	S	R	0.06	0.12	0.25	0.5	1	2	4	8	16	32	64					
Chloramphenicol	≤8	≥32						76.5	21	1.2	0	1.3		2	4	9 (1.2%)	8 (1.71%)	1 (0.34%)

Ciprofloxacin	≤1	≥4	7.2	0.4	0	0.1	0	1.9	90.4					4	4	696 (90.4%)	424 (90.79%)	272 (93.15%)

Erythromycin	≤0.5	≥8				72	8.5	1.2	0.4	0.3	0.5	17.1		0.5	32	139 (18%)	29 (6.20%)	110 (41.98%)^*^

Gentamicin	≤4	≥16		39	14.3	31	13.3	1.6	0.2	0.3	0.3			0.25	1	2 (0.3%)	2 (0.42%)	0

Nalidixic acid	≤16	≥32						6.6	0.7	0.7	1.6	8.4	82	64	64	697 (90%)	428 (91.64%)	269 (92.12%)

Streptomycin	≤2	≥8					66.5	18.2	14.3	0.4	0.6			1	4	8 (1%)	7 (0.14%)	1 (0.34%)

Tetracycline	≤4	≥16			33	1.3	0.2	0.5	0.2	0.8	64			16	16	492 (64%)	272 (58.24%)	220 (75.34%)^**^

S = sensible, R = resistant.

^*^Statistically significant versus *C. jejuni* group (*P* < 0.05, *χ* ^2^ test), ^**^statistically significant versus *C. jejuni* group (*P* ≤ 0.01, *χ* ^2^ test).
